# Sailing global health initiative ships into stormy seas: navigating the introduction of the Global Financing Facility in Mozambique

**DOI:** 10.1080/16549716.2025.2518651

**Published:** 2025-06-26

**Authors:** Andes Chivangue, Mary V. Kinney, Damiano Stella, Denise Namburete, Asha S. George

**Affiliations:** a N’weti, Maputo, Mozambique; b School of Public Health, Faculty of Community and Health Sciences, University of the Western Cape, Cape Town, Bellville, South Africa; c Global Surgery Division, Department of Surgery, University of Cape Town, Observatory, South Africa; d Cooperation and Development Network, Pavia, Italy

**Keywords:** Jennifer Stewart Williams, Global Financing Facility for Women, Children and Adolescents: Examining National Priorities, Processes and Investments, Mozambique, Global Financing Facility, health financing, external financing, policy analysis

## Abstract

**Background:**

Mozambique joined the Global Financing Facility (GFF), a financing mechanism to accelerate progress for women, children, and adolescents’ health, with a history of donor dependence, distrust in public finances, and social inequities. Few independent studies have assessed the GFF.

**Objectives:**

To understand how a global mechanism, such as the GFF, was introduced and utilized in Mozambique.

**Methods:**

This qualitative study explored the aid coordination dynamics between 2015 and 2020 linked to the development of the Investment Case (IC) and Project Appraisal Document (PAD), key national GFF planning documents, based on data from 25 documents and 14 qualitative interviews thematically analyzed.

**Results:**

The GFF was not fully understood by stakeholders and initially gained traction in the name of strengthening the health system, ironically amidst prevailing distrust of government systems. Some viewed the IC as consultatively developed, aiding the Ministry of Health in prioritizing issues and convening donors, while others remained sceptical about its impact. The PAD was viewed as a less consultative process, though it engaged the government and partners in setting disbursement-linked indicators (DLIs) to incentivize health system improvements. However, some stakeholders viewed them as unfeasible, while others were excluded by technical discourse. The perceived transparency issues around DLIs fuelled scepticism.

**Conclusion:**

Although the GFF policy processes provided a technically alluring basis for addressing Mozambique’s health disparities, respondents revealed nuanced perspectives about how IC and PAD were formulated and followed. Aid coordination reflects various interdependencies, power dynamics, and uncertainties that require active relationship management and long-term institution building.

## Paper Context

- **Main findings:** The Global Financing Facility policy processes in Mozambique enabled the convening of key stakeholders and a structured framework for addressing health disparities; however, it was shaped by complex contextual factors, stakeholder skepticism, and challenges in aid coordination.
- **Added knowledge:** This study examines the development and implementation of national planning documents for the Global Financing Facility, a global health initiative, revealing different viewpoints regarding their consultation process, benefits, impacts, and transparency.
- **Global health impact for policy and action:** Effective aid coordination in this context requires the intentional engagement of all stakeholders, including citizens, active relationship management, and long-term institution building.

## Background

Hosted at the World Bank, the Global Financing Facility (GFF) launched in 2015 as a financing engine to accelerate progress for women’s, children’s and adolescents’ health in low- and middle-income countries (LMIC) [1]. Under the banner of a ‘country-led’ approach, GFF-selected countries are meant to follow an inclusive process to outline priorities, strategies, and financial plans for improving reproductive, maternal, newborn, child, and adolescent health (RMNCAH) [2]. Key GFF policy documents include the ‘Investment Case’ (IC), which outlines nationally identified priorities, and the related ‘Project Appraisal Document’ (PAD), which represents the World Bank project loan, whose investments are catalysed by the GFF grant. Since its launch, only a few independent studies have assessed the functions of the GFF process functions [3–6].

Mozambique joined as one of the first countries to be involved in the GFF in 2015 [7]. Mozambique has achieved substantial reductions in maternal, under-five, and neonatal mortality in the past three decades; however, ongoing geographic and social inequities and instability need to be addressed to maintain progress [8]. Various challenges have hindered women’s and children’s health, including limited healthcare infrastructure, scarce health workers, inadequate funding, and low education levels, combined with harmful cultural and societal norms. Healthcare services are mainly delivered by public sector facilities, although private sector providers exist, mostly in urban areas [9].

Mozambique’s history is marked by colonial rule, independence struggles, civil war, and continuous efforts toward peace and stability. Post-independence, the socialist desire for egalitarian health and free universal access experienced successive crises, weakening the credibility of the state despite considerable efforts [10,11]. Alongside neo-liberal reforms and structural adjustment [12], the ruling party centralized power, and by maintaining relative stability, held itself largely immune from calls for further democratic and transparency reforms [13]. In 2022, Mozambique ranked as the sixth largest recipient of foreign aid in Africa [14]. External funding has supported more than 60% of the state’s general budget for over 40 years [15], while significant development challenges remain outstanding [16]. Challenges of aid dependency and donor influence have long been studied and recorded in Mozambique [17,18].

Specific to the health sector, the Health Sector Social and Economic Plan (in Portuguese, known by its acronym PESS) guides national health services [19]; however, several other instruments coexist alongside the PESS for RMNCAH [20], leading to a fragmented policy landscape. Additionally, the multiplicity of development partner agendas has aggravated problems of coordination, challenging the Ministry of Health’s (MOH) management and leadership role [19–23]. To address this fragmentation, a sector-wide approach (SWAp) was established in 2000 to enable a holistic and coordinated approach between donors and the government [20]. In 2003, PROSAÚDE (the Health Common Fund financed by several multilateral and bilateral development partners) became the SWAp backbone [20,24]. However, contributions to the PROSAÚDE Common Fund began declining in 2013, reflecting both shifting domestic priorities in donor countries to cut back on aid and growing distrust of Mozambique’s political governance. These concerns have been fuelled by a trend toward democratic re-centralism and enduring elite patron–client relationships, which have undermined donor confidence and commitments [25].

With this history of donor dependence, distrust in public finances, and continued gaps for RMNCAH, Mozambique presents a valuable case study for describing how a global mechanism, such as the GFF, gets introduced and utilized by governments facing multiple challenges in advancing their health priorities. Specifically, this study focuses on the development of the two main GFF policy documents, IC and PAD. In undertaking this policy analysis, we aim to identify and understand the factors that shaped the content of these GFF country documents in order to inform future policies and processes related to GFF and other global health initiatives. The study was done in conjunction with the ‘Countdown GFF policy analysis collaboration,’ a multi-disciplinary group of academics and partners working in the African region [26], who led similar in-depth case studies in Burkina Faso [27], Tanzania [28], and Uganda [29].

## Methods

### Study design

This qualitative case study seeks to explore the GFF policy processes in Mozambique retrospectively, between 2015 and 2020, when the GFF was introduced, and some initial implementation was undertaken. This study seeks to describe the process of developing GFF policy documents, considering the actors involved, governance structures, official and unofficial processes, and the context of the setting.

### Data collection and management

Data collection involved a desk review and key informant interviews. The desk review identified 29 documents including the GFF policy document, government policies, policy and research briefs, donor reports, and journal articles (Table 1, Supplementary file 1). For the qualitative interviews, we purposively selected and contacted 22 respondents by email who had been instrumental in the development of IC and PAD. Fourteen individuals were interviewed (Table 2). We reached out to those who worked for donor agencies, including bilateral and multilateral organization (i.e. UNICEF, WHO, UNAIDS, UNFPA), government, including different departments, such as ministries of finance and health, and other actors who were engaged in the process, such as those who represent civil society and academia. Interviews were conducted in Portuguese, remotely, or face-to-face, depending on the respondent’s preference, using a semi-structured interview guide after obtaining consent. Interviews were undertaken by AC, a political scientist with extensive experience in the health sector, who adapted the interview guide based on respondent profiles and to follow triangulation and saturation principles. Each interview lasted for an average of 45 min to 1 h and took place in a location that the respondent suggested (mostly their offices). The recorded interviews were transcribed verbatim into a word file and then translated into English. Data were collected between November 2022 and February 2023.

**Table 1.** Documents reviewed.

Type	Number	Examples
Official policy documents	11	Health Sector Strategic Plan (PESS 2014–2019); Investment Case, PAD; PAD restructuring, Service Delivery Indicators, Health Financing Strategy, National Health Policy, Mid-term review, World Bank reports (3)
Scientific data/evidence	11	Journal articles (7), Book section (1), Surveys and published reports (3)
Grey literature	6	Research brief (2); Policy briefs (4)
Other	1	Webpage (1)
TOTAL	29

**Table 2.** Details of respondents.

Respondent type	Total contacted	Interviewed
Government	6	3
Donors (World Bank, bilaterals, multilaterals)	10	8
Other actors (CSOs, academia, consultants, technical assistance)	4	3
Total	20	14

### Data analysis

Four analysis tables and a coding framework based on the health policy triangle [17] were co-designed with a broader study collaboration and based on emergent themes [26]. The interviews were initially coded and analyzed manually by AC, who produced a summary table for each interview, and then coded by MVK using Atlas.ti v24. Summary tables were discussed further by the author team. A power-position grid analysis was conducted to understand stakeholder dynamics in the GFF policy processes [30]. Members of the Countdown GFF policy analysis collaboration, including AC, MVK, and AG, participated in two face-to-face workshops (October 2022 and March 2023) and met weekly for 1 h from October 2022 to March 2023 with the broader collaboration to co-develop the analysis tools, reflect on the data collection and analysis process, and debrief on emerging themes.

### Positionality, reflexivity, and ethics consideration

Two authors (AC (male) and DN (female)) worked for N’weti, a Mozambican non-profit organization, which conducted an earlier investigation on GHIs [3]. As advocacy organizations, they support civil society oversight efforts that aim to hold the government and bilaterals accountable. DS (male) worked as a Public Financial Manager Advisor to the Health Sector, aiming to improve the sector’s capacity for policy-based budgeting and strategic dialogue on Health Financing. MVK (female) and AG (female) are based in South Africa but bring global experience of understanding GFF policy, with AG having collaborated on previous policy analyses in Mozambique.

Ethics approval was received from the University of the Western Cape’s Biomedical Ethics Review Committee and the National Health Commission for Bioethics. The study is in accordance with the principles of Declaration of Helsinki. All participants provided informed verbal or written consent and were assured of the confidentiality of their responses, including anonymity, during the dissemination of findings. Several measures were implemented to ensure research rigor: engaging stakeholders prior to data collection, ensuring voluntary participation, obtaining peer and expert feedback, maintaining an audit trail with clear mapping of the research process, and triangulating data sources. Preliminary results were fed back to the Mozambique Ministry of Health and the Global Financing Facility (GFF) secretariat to validate the results.

## Results

**Figure 1.**
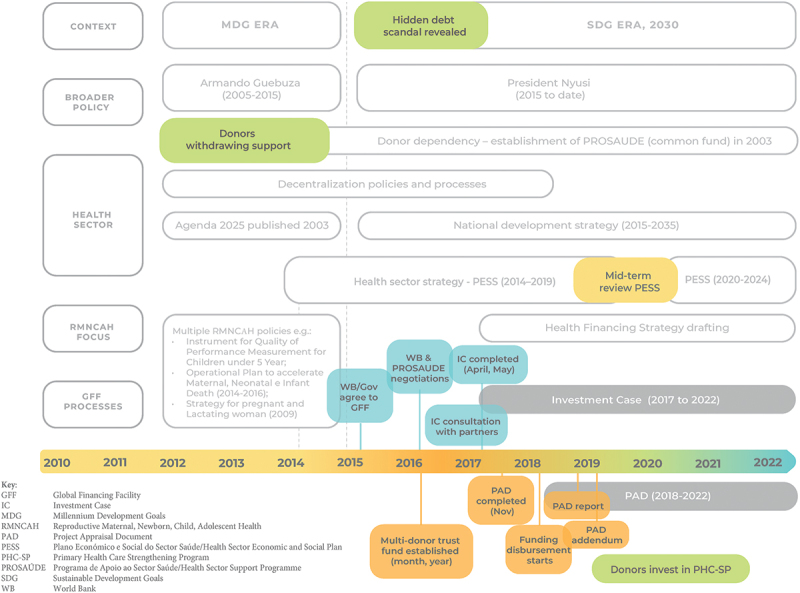
Policy and process timeline relating to the GFF country documents.

The GFF process in Mozambique began under government leadership and in collaboration with a range of partners in 2015 (Figure 1). The GFF policy documents (IC and PAD) set out priorities, plans, indicators, and expenditures for a five-year period from 2017 to 2022 (Table 3), addressing RMNCAH issues prioritized in the broader PESS and National Health Policy. IC is a broad-costed implementation strategy with a proposed budget of US$1.8 billion [31]. The PAD includes the GFF multi-donor trust grant and focuses on improving the utilization and quality of RMNCAH and nutrition in underserved areas with a specific investment of US$105 million through the project called the ‘Primary Health Care Strengthening Program’ (PHCSP) [32]. It also includes contributions from the ‘multi-donor trust fund’ established for the PHCSP; reported commitments from PROSAÚDE, the Netherlands, and USAID; interest from the Canadian Department of Foreign Affairs, Trade and Development (DFATD); and support for the IC from the UK Department for International Development (DFID) and UN agencies.

**Table 3.** GFF planning document contents.

	IC	PAD
Overview	The IC built on the PESS	Further prioritised areas identified in IC
Dates	5 years (2017–2022), published April 2017	5 years (2018–2022), published November 2017
Mindset	Priority topics: MNH, reduction in fertility, Identifies focused geographical areas	Primary Health Care – Strengthen Programme with focus on specific districts in selected provinces with a performance for results framework embedded in it
Measures	Comprehensive results framework	11 Disbursement Linked Indicators 6 Program Development Objective results indicators
Money	Total: US$1,837 M	Total US$105 M● IDA: US$80 M ● GFF: US$25 M
		Other contributors listed in PAD:●PROSAUDE: US$16 M ● Netherlands: US$35.5 M ● USAID: US$22.5 M ● Borrower (government): US$963 M

While the health sector remains aid-dependent, health expenditure has been increasing, with increases in development assistance and government spending in the past decade (development assistance increased from US$605 million in 2015 to US$1.05 billion in 2021; government spending increased from US$273 million to US$467 million, respectively) [33]. Country-specific official development assistance to RMNCAH in Mozambique decreased slightly from US$254 million in 2015 to US$238 million in 2019 [34]. Prior to GFF, domestic resources specific to RMNCAH were not tracked [35]. It is within this context that GFF was introduced to support financing for RMNCAH.

Several contextual factors, as discussed below, were highlighted by respondents as contributing to how the GFF policies developed and the dynamics involved. In the next sections, we review in more detail the ebbs and tides of the IC and PAD development processes in Mozambique.

### Initial negotiations and window of opportunity

The development of GFF country documents in Mozambique started in 2015 with various consultations and discussions between the Government, World Bank, development partners, and relevant stakeholders. The GFF, within the World Bank and in partnership with the MOH, convened various meetings and set up oversight structures in an effort to sensitize stakeholders within and outside the government about the GFF opportunity. These were new working groups because the formal structures for government dialogue with other partners were not functioning at the time. Partners provided technical support to support the development of the GFF policy documents. For example, UNICEF supports the use of the Equist tool to inform IC priorities. However, respondents had mixed perspectives on stakeholder engagement and the consensus-building process.

Part of the challenge was that initially, there was a misunderstanding of what the GFF entailed in terms of resource mobilization (Respondents 4, 6, 8, 13). When the GFF was first introduced, the World Bank did not fully describe the functions of the GFF and planned architecture.

It is clear that this financing instrument was completely new for most people, especially not only by the Ministry of Health but also by the partners themselves (Respondent 13)

The Bank was never clear on the distinction between the PAD and the IC. Most of the actors thought that it was the same and the Bank would finance everything (Respondent 6)

In addition to this confusion, there was a lack of clarity about how the new funding mechanism would fit within an already established mechanism, notably PROSAÚDE [11]. Discussions were held with PROSAÚDE to include the GFF and create a hybrid fund; however, consensus was not reached among the partners involved (Respondent 13). Subsequently, there were perceptions of competition between PROSAÚDE and the new multi-donor approach for the PHCSP (Respondents 2 and 4) and a lack of consensus across development partners and within the MOH about which to support (Respondent 7).

While the GFF promised to catalyse further funding in the context of declining resource mobilization, some saw it as an overly narrow approach to RMNCAH, not capable of strengthening the health system as a whole. Many ministry personnel preferred PROSAÚDE due to its history as a common fund and its embeddedness within the national planning and budget processes [11]. However, underlying structural limitations in the national budgeting system – such as the absence of policy-based or program-based budgeting and reliance on historical input-based allocations – may have constrained the strategic use of any external funds, including PROSAÚDE (Respondent 1). Some respondents felt that these systemic weaknesses contributed to dissatisfaction and scepticism about the effectiveness of PROSAÚDE, based on audits [36].

Against this backdrop, unprecedented corruption at the highest level of government-led development partners to suspend direct aid in 2016 [37]. This hidden debt scandal came to light in a moment when government and donors in the health sector were already grappling with transparency on procurement, delays on releasing the auditing reports and claims of financial mismanagement [38]; as such, government and donor personnel in country needed to show a different modality of funding for health. Some respondents felt that the GFF became a perceived alternative funding mechanism for donors given concerns over mismanagement and transparency heightened by the debt scandal (Respondents 3, 4, and 13). The GFF gave the impression of something new and an alternative modality of funding while keeping or increasing the level of disbursement for Mozambique.

The hidden debt revelation brought distrust and donors felt that it was not safe to channel money through national systems. (Respondent 13)

The debt crisis broke out and this path was completely blocked … so the alternative that emerged was the GFF … knowing that the money would be made available to the Ministry of Health, to implement activity A,B,C,D,E, so this was judged to be an attractive option. (Respondent 4)

Some donors came on board, while others resisted it. Those who decided to join the multi-donor trust fund were attracted by the World Bank’s capabilities to deal with fiduciary risks and administration, including its perceived auditing capability.

The perception that some had was that the bank, or its advantage of exercising fiduciary control, would be in a better position to direct these funds to be invested in the health sector and minimise the risks in terms of deviations from application. In fact, one of the partners thought that the bank should exercise all fiduciary control (Respondent 13)

Some respondents also reported an overall perception of the government not being able to lead policy implementation effectively, partly due to the fragmentation of the health system and the challenges and transaction costs involved in coordinating development assistance. Formal policies and expertise existed, but this did not translate into coordinated action.

The absence of a strategy in a real sense represents a problem. The sector knows where it wants to reach but it does not know how to do it (Respondent 1)

There is no real planning in Mozambique. The PESS is not linked to what donors do nor to what the government does (Respondent 2)

Despite existing scepticism toward common approaches to health-sector financing, the GFF process advanced rapidly. This urgency was largely driven by tight timelines to finalize the PHCSP through the World Banks’ PAD, which forced partners to move faster in policy negotiations. At the same time, PROSAÚDE was working on its third Memorandum of Understanding and was unable to keep pace with the speed at which the IC and PAD were progressing (Respondent 7).

The World Bank pushed the IC to be the centre of any policy and dialogue in the health sector, which would replace any policy for the domain of maternal and child health (Respondent 12)

Once the PHCSP was established, many donors joined the multidonor trust fund (at least initially). Some donors perceived it as ‘the only game in town’ for RMNCAH (Respondent 2). Additionally, the host organization of the GFF within the World Bank, was perceived as the “gorilla in the room’ (Respondents 4, 5, and 10) because of its financial power and administrative capability. Thereafter, the GFF gained traction, even if it was not fully understood, in the name of government health systems strengthening (Respondent 5), ironically amidst prevailing distrust of government systems.

## IC development and use

Given the misunderstandings and the varied reactions from different partners when the GFF opportunity was announced, meetings were held to make sure that all stakeholders were on the same page (Respondent 13). For IC drafting, the majority of the fortnightly sessions were held in the MoH led by the National Directorate of Public Health (Respondents 4, 13), with a balanced division of labour among development partners (Respondent 6). Interactions supporting the drafting of the IC were viewed positively, with progress reviewed at the Health Partners Group’s monthly meetings (Respondent 4). Consultations were also held with beneficiaries such as adolescents, professional associations, and the private sector (Respondent 13).

The IC was reportedly internally approved by the MOH and endorsed and accepted as the document behind which all partners aligned their financing (Respondent 2 and 4). Those supporting the multi-donor trust fund saw the IC as crucial in helping the MoH focus on the interventions needing more attention and resources (Respondent 6, 13). Nonetheless, confusion about the purpose and use of the IC remained even at the time of data collection for this study, 5 years after it was introduced. There was acknowledgment that the IC's purpose was mainly to build a consensus among development partners and may not have led to actual changes by the government.

The IC is not a legal binding document, it is just an analytical input to design PAD and the project. … .I think this is also a reflection of a lack of understanding of the notion of an investment case … (Respondent 4)

Despite the positive reflections of some respondents, others remained critical. The MoH managed different versions of the document, resulting in no final agreed version [31]:

No one had access to the final draft of the IC, nobody knew if it was finished and the word IC became a ‘cursed’ word … The dialogue is superficial, the documents are not widely shared, and the themes discusses are not those of interests of all; The MoH is not engaged as required and nothing has changed. (Respondent 12)

## PAD development and implementation

PAD developed after the IC under the leadership of the World Bank (Figure 1). The timeline was the first thing that was negotiated with the Government, as the World Bank did; therefore, GFF-linked financing had a preset schedule for World Bank board approval. A specific session was held with the MOH to ensure that multi-donor trust fund financial resources would not result in crowding out of government financing. Technical guidance was prepared accordingly in consultation with the Ministry of Financing through sessions led by the MOH Directorate of Planning and Cooperation.

The PAD project, or PHCSP, used the Performance for Results funding instrument governed by disbursement-linked indicators (DLIs). Respondents had mixed views on this approach in terms of how it was framed by the World Bank and the capacity of the government to implement it (Respondent 1). Some donors look at the Performance for Results element as the best way of financing the health sector and the learning curve to understand it, on part of the government and other partners, as normal (Respondent 9).

There was not always 100% consensus on the type of instrument, but the idea that existed was that results in neonatal maternal reproductive health, etc., would only be achieved if the program contributed to some paradigm shift (Respondent 13)

DLIs were seen as critical to incentivize results, and technical groups led by relevant figures in the MOH were constituted to elaborate them. Partners were invited to join these groups, where they had a comparative advantage (Respondent 13). Other government sectors were also involved, e.g. the Ministry of Education for school health and the Ministry of Justice for vital statistics and civil registration. However, various tensions arise during PAD development. Some partners disagreed with the defining of the DLIs (Respondent 5) and the technical nature of the processes also made some feel excluded, particularly some donors and civil society group.

The PAD is a very strange document with a level of complexity which makes the reader feel not smart enough to understand it (Respondent 5)

The discussion were very technical which excluded some of the partners (Respondent 7)

While the drafting of the PAD initially benefited from the participation of both bilateral and multilateral partners, as well as a small group of civil society organizations, the extent of this consultation was also reflected as incomplete.

Now, it probably could have been better in terms of the variety of civil society organizations participating in this process … These consultations took place, but perhaps they could be more extensive, they ended up participating practically the same organizations and perhaps could have been a little more comprehensive (Respondent 13)

While several donors were initially part of the PAD, their positions continued to evolve. Delays in PHCSP implementation lead some partners, such as USAID, to leave the PHCSP and establish a government-to-government (G2G) mechanism, enabling them to directly fund at the district level (Respondent 13). However, some partners also felt that they were not part of the discussions and decisions around implementation, particularly those related to the performance of results programming.

If fact, there was an expectation that by investing in PHCSP the financier would have a place around the table in the discussions of policies, but this discussion was monopolized by the World Bank. (Respondent 4)

Although the GFF initiative gained traction initially as an alternative mechanism to support the health sector due to concerns about government capacity and accountability, similar concerns and confusion arose with initial implementation of the DLIs (Respondent 5).

The DLIs are seen as the proper instrument to introduce incentives but the ministry does not know how to do it. This explains why in the first years the execution rate was minimum (Respondent 1)

To ensure accountability of the PHCSP, community score cards were used and made available by one of the CSOs implementing them. However, the results of the Performance Card – managed by the MOH, were not publicly available, which, as one respondent stated ‘gives the impression that there are intransparent things happening inside’ (Respondent 7). Another respondent noted

In some cases the intended results were changed … to allow funds disbursement (Respondent 4)

This has resulted in unintended consequences of implementing DLIs in a health system in which existing problematic power dynamics remain unaddressed.

Data is often used for accountability, in the sense punishing for failures. And this approach is also externally induced by donors. This leads to a situation were data is not produced or disclosed (Respondent 1)

## Comparing the IC and PAD processes

As per the GFF’s intention to support countries in coordination, priority identification, and planning for implementation of interventions, we found that the processes varied greatly between the two policy documents. Generally, the IC process was perceived as inclusive and transparent, whereas the PAD process did not engage stakeholders in the same way, even though the GFF was linked to the PAD. Those outside the process did not feel that there was legitimate consensus building, understanding, and buy-in, particularly on the performance-based financing approach in the PAD. The DILs introduced in the PHCSP were designed to give a purpose to the investment of funds and provide the incentive for this change. However, DLIs turned into a complex mechanism for releasing funds, with little evidence of changing the modality of planning and budgeting by government.

Stakeholders identified as critical to the processes included representatives from the Ministry of Health’s Planning and Cooperation Directorate, Public Health Directorate, and department heads; World Bank program leaders and specialists; donors including GIZ, USAID, DFID, Flanders, and Canada; technical consultants; representatives from UN agencies; and civil society actors. However, a stakeholder power-position mapping shows that only government and donor stakeholders were involved in both processes and that donors had the greatest influence and highest interest in ensuring GFF policy success (Supplementary file 2).

This may explain why some respondents claimed that decisions agreed among partners and the Ministry were later changed by the World Bank in bilateral negotiations with the government.

Some of decisions agreed amongst partners and the Ministry regarding the technical notes were unilaterally changed by the World Bank (Respondent 1)

The investment case was prepared in a very transparent way and when it comes to the preparation of the PAD and the Performance for Results this happened unilaterally, where the government was, I think, consulted, being part of that process of project development under the World Bank. But everyone else on the outside wasn’t around, then this project developed the way it did, there were some consultations, things like that, check box exercise I can say it like that, but in concrete terms we didn’t have the desired level of involvement or of dialogue (Respondent 4)

The policy processes were also challenged by the level of turnover among government personnel in the health system.

We’ve had a lot of changes over time since the year we started designing the project until the time it was approved until now it’s being implemented, therefore, it is quite normal for me to … be with a colleague who I have never heard of or is not very aware of because of this change of staff at the health sector level, unfortunately it is a reality that we have. (Respondent 11)

## Discussion

This paper presents original qualitative evidence on how the GFF was perceived, negotiated, and implemented, marked by a stormy context of aid dependency, longstanding donor and other stakeholder coordination challenges, and ongoing political instability and centralization. While mechanisms for aid coordination preceded the GFF, the confirmation of a massive corruption scandal in 2016 provided a window of opportunity for the GFF to present itself as a way of doing business differently, with promises of more strategic investment and stronger fiduciary control. While the IC was developed more inclusively than the PAD, both policy documents had elements of collaborative input from various development partners. Although both the IC and PAD provide a technically alluring basis for addressing Mozambique’s health disparities, respondents revealed nuanced perspectives on how these documents were formulated and followed. Some respondents continued to affirm their strategic importance while others remained sceptical, and some donors walked away from the process.

Mozambique has historically been highly dependent on foreign aid and is no stranger to positive, albeit lapsed, experiences of aid coordination [18,20,39]. Critical to understanding the stormy contexts in which the GFF was introduced and its ability to effectively marshal other donors is the country’s broader political economy. While the MoH led the GFF policy development process, its role has historically been constrained by structural adjustment policies that stymied its ability to accept foreign aid as part of the general budget support [12]. While this was not the only reason why donors were funding the health sector independently of the government, it played a critical role in undermining the government’s ability to deliver on its governance mandate [20]. More critically, some have noted that the government of Mozambique has depended on foreign aid to fund services, while it has reserved resources from extractive industries for its own wealth accumulation and maintained stability with increasing centralization and authoritarianism [40,41].

It is within this broader political storm that stakeholders in Mozambique were required to coordinate and deliver on the GFF policy documents. In comparison to the other three country studies in this Collection [26], we found some similarities. In Burkina Faso, where political instability and insecurity affected the process, unclear government leadership, high turnover in key actors, and donor reluctancy and mistrust in the GFF processes were also identified as challenges [27]. However, in Uganda and Tanzania, which had more politically stable environments, it was also found that the GFF policy process was deeply negotiated spaces, and the PAD processes less transparent [28,29]. The role of power shaping policy dialogues, particularly donor influence, has been found to be both positive and negative in other African countries [42]. Government ownership and donors’ influence may successfully coexist at various stages in policy processes; and these actors may be working together strategically when navigating complex environments [43], such as the one in Mozambique when GFF was initiated. Aid coordination reflects various interdependencies, power dynamics, and uncertainties, which have long been documented in Mozambique specifically [17,23]. Even with consensus building and coordination milestones, these can be undone as government and donor agency personnel move on and contexts and understandings change. Lessons from past experiences from SWAPs recommend moving away from blueprints to modalities where stakeholders can iteratively learn how best to work together over time through continued dialogue and trust-building [22]. However, this may be less possible in contexts with high staff turnover, further inhibited by clouds of state capture by the national elites.

Supporting platforms for discussing financing commitments and modalities could help to better understand stakeholder perceptions, clarify confusion, and develop common ground – ensuring people are at the centre of health financing [44]. Our analysis highlights two conflicting narratives among the respondents. Some donors and some in the MOH were eager to agree to the GFF processes to overcome the health-sector financing crisis in a way that provided more perceived credibility than previous modalities. At the same time, there was also a group of donors who manifested scepticism, fearing loss of visibility, and direct access to the MOH. From the MOH perspective, resistance was closely linked to the fear of a new fragmentation of health-sector financing. We also found confusion around the GFF functions, especially when it started. The lack of publicly available guidance until 2019 meant that stakeholders had little clarity about what the GFF was or how it would operate in practice [2]. Importantly, it was and remains unclear who the GFF is ultimately accountable to – raising questions about legitimacy, responsiveness, and the balance of power between global and national actors.

In a new report, the World Bank argued that public participation is key to fairer processes for financing universal health coverage [45]. Fair processes, including public participation, help to understand the views of under-represented groups, build legitimacy and trust, and support more sustainable foundations for financing decisions. In the Tanzania GFF policy analysis, there was a sense of closed spaces preventing meaningful engagement with civil society through the process [28], whereas in Uganda, civil society engagement was able to counterbalance demands from national elites in determining financing priorities in the GFF process [29]. However, McCoy (2025) [46] cautioned against the simplistic assumption that public participation necessarily prioritizes equity. Further research is needed to better understand the contexts and conditions through which public participation and transparency strengthen the prioritization and accountability of funding mechanisms designed to support national health priorities.

Since our data collection, the World Bank approved a new PAD investing US$100 million and US$15 million from the GFF on 6 February 2024. While the agreement was developed more consultatively and CSO participation was supported by the MOH, it was delinked from monitoring the Community Score Cards in performance-based financing. Furthermore, rather than referring back to the IC, a health sector financing strategy, a complementary framework to the IC, was approved. While aiming to increase health-sector financing, navigating the complexities of donor coordination amidst open political contestation reaffirms the importance of institution and relationship building with a broad stakeholder base to weather storms.

A number of research priorities emerge from this study. First, there is a need to better understand which models of inclusive and transparent priority-setting can enhance the legitimacy and practical relevance of investment cases in low-resource settings. Second, further research is warranted on the institutional reforms required to align global health financing mechanisms – such as the GFF – with existing country systems to improve their effectiveness and sustainability. This is particularly relevant in Mozambique, where funding modalities remained largely unchanged despite GFF being presented as something new. Finally, greater insight is needed into how national public financial management systems enable or constrain the strategic allocation of external health financing.

## Strengths and limitations

Our analysis is based on data collected in 2023 and is primarily led by those outside the government and development partner circles. While we documented various nuances, the dynamic and largely closed nature of donor government-financing negotiations made our work challenging. Not all high-level policy stakeholders involved in the process were interviewed, despite attempts to reach them out. Some of the identified stakeholders had left their positions, and some were no longer in the country and did not respond to our interview requests. We acknowledge that the small number of individuals interviewed was a limitation. To address this, we ensured that the desk review was comprehensive and inclusive of policy documents as well as academic literature. We triangulated the information from the desk review, and key informant interviews to draw out common themes. We also shared preliminary results with key stakeholders, including the MOH and the GFF secretariat, to enable input from a wider range of individuals involved in the processes. Their feedback was considered and incorporated, as appropriate. The embedded nature of Mozambique-based authors committed to health system improvement combined with those with expertise from other contexts helped strengthen this paper.

## Conclusion

This paper presents a country-level analysis of the GFF policy process in Mozambique, offering new insights into how global health initiatives interact with complex national governance processes. Although the IC and PAD provide a technically alluring basis for addressing Mozambique’s health disparities, especially given concerns about government capacity, respondents revealed nuanced perspectives on how these documents were formulated and followed. While some continued to affirm their strategic importance, others remained sceptical, and some donors walked away from the process. Aid coordination in this context reflects a complex interplay of interdependencies, power dynamics, and uncertainties. Supporting platforms that enable open communication, relationship building, trust, and mutual understanding are critical parts of institution building needed to weather the uncharted stormy financing waters that lie ahead.

By tracing the evolution and initial implementation of the IC and PAD within a broader history of aid coordination and public financial management, the analysis underscores how institutional constraints and historical relations can influence the intended catalytic function of new funding mechanisms. The findings highlight that the success of global financing initiatives depends not only on their technical design but also on the governance context into which they are introduced. There is a need to strengthen country capacities not only to receive funds but to strategically allocate and align them within coherent, accountable systems.

## Supplementary Material

Supplementary_files_PRE EXPORT.docx

COREQ_Checklist Mozambique.pdf

## Data Availability

The datasets used and/or analyzed in this study are available from the corresponding author upon reasonable request. This paper is part of the *Global Health Action* Special Series, ‘Global Financing Facility for Women, Children and Adolescents: Examining National Priorities, Processes and Investments’ available in Volume 17–01.
