# Supplementary material for: Sailing global health initiative ships into stormy seas: navigating the introduction of the Global Financing Facility in Mozambique
Source: Glob Health Action. 2025 Jun 26;18(1):2518651. doi: 10.1080/16549716.2025.2518651 (PMC12203692; doi:10.1080/16549716.2025.2518651)

Supplementary files

## Supplementary file 1: Documents identified and included in the review

|  | Document name | Authors | Publication Date | Reference / web link | Type of document | Category for paper |
| --- | --- | --- | --- | --- | --- | --- |
| 1 | Annual Health Sector Balance Report 2020/ *Relatório Anual de Balanço do Sector de Saúde 2020* | MISAU | 2020 |  | Mid-term review | Official policy documents |
| 2 | Relatório de Avaliação do Plano Estratégico do Sector de Saúde (PESS 2014-2019) | Ministéro da Saúde (MoH) | 2019 | Assessment report (Relatório de Avaliação) | Mid-term review | Official policy documents |
| 3 | Mocambique: Programa de Fortalaecimiento de Cuidados de Saude Primarios; Missao de Revisao para a Restructuracao | World Bank | 2020 | Documents relating to project: https://documents.worldbank.org/en/publication/documents-reports/documentlist?qterm=P163541 | World Bank - PAD | Official policy documents |
| 4 | PESS - Plano Económico e Social Sector Saúde 2014-2019 | Direcção National de Planificação e Cooperação (National Directorate for Planification and Cooperation - MoH) | 2013 | https://extranet.who.int/countryplanningcycles/sites/default/files/planning_cycle_repository/mozambique/mozambique_-_health_sector_strategic_plan_-_2014-2019.pdf | Policy | Official policy documents |
| 5 | Mozambique Primary Health Care Strengthening Program-for-Results (P163541) | World Bank | 2017 | <https://documents1.worldbank.org/curated/en/443651513005902836/pdf/MOZAMBIQUE-HEALTH-PAD-12012017.pdf> | World Bank - PAD | Official policy documents |
| 6 | Proposta de Resolução – Política Nacional de Saúde. Unidade da Reforma: Maputo. | MISAU | 2020 |  | Policy | Official policy documents |
| 7 | Strategic Guidance Document Health Sector Financing 2020-30 / Documento orientador estratégico Financiamento do Sector Saúde 2020-30 | MISAU | 2019 | https://p4h.world/app/uploads/2023/02/Strategic20guidance20document20on20Health20Sector20Financing202020-203020in20Mozambique.x23411.pdf | Policy | Official policy documents |
| 8 | *Plano Estratégico do Sector da Saúde (PESS) 2014-2019 (2024)*. | MISAU | 2025 | https://p4h.world/wp-content/uploads/2014/06/2016_06_06_MISAU-HFS_DRAFT3.x80726.pdf | Policy | Official policy documents |
| 9 | MOÇAMBIQUE: Programa de Fortalecimento de Cuidados de Saúde Primários; Missão de Apoio a Implementação | World Bank | 2018 | Documents relating to project: https://documents.worldbank.org/en/publication/documents-reports/documentlist?qterm=P163541 | World Bank - PAD | Official policy documents |
| 10 | Mocambique: Programa de Fortalaecimiento de Cuidados de Saude Primarios; Missao de Apoio a Implementação | World Bank | 2019 | Documents relating to project: https://documents.worldbank.org/en/publication/documents-reports/documentlist?qterm=P163541 | World Bank - PAD | Official policy documents |
| 11 | MOÇAMBIQUE: Programa de Fortalecimento de Cuidados de Saúde Primários; Missão de Apoio a Implementação | World Bank | 2021 | Documents relating to project: https://documents.worldbank.org/en/publication/documents-reports/documentlist?qterm=P163541 | World Bank - PAD | Official policy documents |
| 12 | Índices das Actividades Económicas Julho 2021. Brochura. Maputo. Instituto Nacional de Estatística. | Instituo Nacional de Estatistica | 2021 | <http://www.ine.gov.mz/estatisticas/estatisticas-economicas/indice-de-actividades-economicas-iae/brochura_iae_no207_julho-2021jr_ch.pdf/view> | Data | Other |
| 13 | INE. 2021b. Inquérito sobre Orçamento Familia (IOF) 2019/2020.Maputo. Instituto Nacional de estatística. Relatorio Final. | Instituo Nacional de Estatistica | 2021 | <http://www.ine.gov.mz/operacoes-estatisticas/inqueritos/inquerito-sobre-orcamento-familiar/iof-2019-20/inquerito-sobre-orcamento-familiar-iof-2019-20/view> | Data | Other |
| 14 | Orçamento proposto para o sector de saúde para 2021 sustenta-se em 81,8% com recursos internos e 18,2% com recursos externos | The Citizen Observatory for Health (OCS) |  | <https://www.observatoriodesaude.org/orcamento-proposto-para-o-sector-de-saude-para-2021-sustenta-se-em-818-com-recursos-internos-e-182-com-recursos-externos/> | webpage | Grey literature |
| 15 | Global Financing Facility (GFF): an alternative health sector financing model? Policy Brief 08 | N'weti | 2019 | https://nweti.org/en/global-financing-facility-gff-an-alternative-health-sector-financing-model/ | Policy brief | Policy briefs |
| 16 | *Analysis of Institutional, Financial and Political-economic Issues*. Policy note 02 | N'weti | 2023 | https://nweti.org/en/policy_brief/financing-the-health-sector-in-mozambique-continuous-shortages-and-inadequate-allocation/ | Policy brief | Policy briefs |
| 17 | Towards a new strong funding mechanism? For the joint work between PROSAÚDE Common Fund and the Global Financing Facility. Policy brief 12 | N'weti | 2023 | https://nweti.org/en/policy_brief/could-prosaude-and-gff-work-together-on-a-joint-funding-mechanism/ | Policy brief | Policy briefs |
| 18 | GFF em Moçambique: o fosso entre aspirações sobre RMNCAH-N e o desafio de realidades institucionais complexas. Policy brief 15 | N'weti | 2023 | https://nweti.org/pub/gff-em-mocambique-o-fosso-entre-aspiracoes-sobre-rmncah-n-e-o-desafio-de-realidades-institucionais-complexas/ | Policy brief | Policy briefs |
| 19 | “Saúde para o Povo? Para um Entendimento da Economia Política e das Dinâmicas da Descentralização no Sector da Saúde em Moçambique”. In: Weimer, Bernhard, (ed.), Moçambique: Descentralizar o Centralismo. Economia Política, Recursos, Resultados. | Weimer, Bernhard | 2012 | https://docs.programapotenciar.com/lib/LRYLIB2W | Book section | Scientific data/evidence |
| 20 | State Budget for Fiscal Year 2020: MOZAMBIQUE Analysis of the Social and Economic Sectors | UNICEF | 2020 | https://www.unicef.org/mozambique/media/2786/file/Budget_Brief_2020_-_Analysis_of_2020_State_Budget.pdf | Budget report | Scientific data/evidence |
| 21 | Health, development, and institutional factors: The Mozambique case. WIDER Working Paper 2020/131. Helsinki: UNU-WIDER. | Garrido, Paulo Ivo | 2020 | <https://www.wider.unu.edu/sites/default/files/Publications/Working-paper/PDF/wp2020-131.pdf> | Journal article | Scientific data/evidence |
| 22 | “The Paradox of Redistribution and Strategies of Equality: Welfare State Institutions, Inequality, and Poverty in the Western Countries”. *American Sociological Review* Vol. 63, No. 5 (Oct., 1998), pp. 661-687 | Korpi, Walter & Palme Joakim. | 1998 | <https://doi.org/10.2307/2657333.> | Journal article | Scientific data/evidence |
| 23 | Assessing the impactof aid on public health expenditure in aid recipient countries | Lim, Yoojin, et al. | 2021 | <https://onlinelibrary.wiley.com/doi/epdf/10.1111/dpr.12635> | Journal article | Scientific data/evidence |
| 24 | Programs of maternal and infant health in Mozambique: Gradual Landmarks and the Insertion of Nursing. |  | 2009 | https://www.scielo.br/j/ean/a/s8PKGHnWW3qCrKkzSgg7QMR/?lang=pt | Journal article | Scientific data/evidence |
| 25 | (2011). “Two-tier charging in Maputo Central Hospital: costs, revenues and effects on equity of access to hospital services.” BMC Health Services Research 11.1 (2011): 1-8. | McPake, Barbara; Charles Hongoro& Giuliano Russo | 2011 | https://bmchealthservres.biomedcentral.com/articles/10.1186/1472-6963-11-143 | Journal article | Scientific data/evidence |
| 26 | Drivers of health system strengthening: learning from implementation of maternal and child health programmes in Mozambique, Nepal and Rwanda | Samuels, Fiona; Ana B Amaya, and Dina Balabanova | 2017 | https://doi.org/10.1186/s12939-017-0673-0 | Journal article | Scientific data/evidence |
| 27 | Interrogating the World Bank’s role in global health knowledge production, governance, and finance | Tichenor, Marlee; et | 2021 | <https://globalizationandhealth.biomedcentral.com/articles/10.1186/s12992-021-00761-w> | Journal article | Scientific data/evidence |
| 28 | Global Financing Facility (GFF): narratives of adherence and implementation dynamics Research biref 02 | N'weti | 2019 | https://nweti.org/en/global-financing-facility-gff-narratives-of-adherence-and-implementation-dynamics/ | Research brief | Scientific data/evidence |
| 29 | Global External Financing Mechanisms of the Health Sector in Mozambique - case studies, institutional, financial and political-economic considerations | Bernhard Weimer | 2022 | <https://nweti.org/en/policy_brief/global-external-financing-mechanisms-of-the-health-sector-in-mozambique/> | Research brief | Scientific data/evidence |

## Supplementary file 2: Stakeholder Power–Position Grid Analysis

### Details on methods

To better understand stakeholder dynamics during the development and implementation of the GFF policy documents in Mozambique, we conducted a power–position grid analysis. This method helps map key actors based on their relative influence ("power") over decision-making processes and their stance ("position") in support of or opposition to the policy initiative.

Stakeholders were identified through document review and qualitative interviews. Each actor or group was assessed using data from interview transcripts and validated through triangulation with policy documents and meeting notes. Actors were identified based on their stakeholder grouping as well as their engagement levels in the IC and PAD processes. Power was defined as the ability to shape decisions, mobilize resources, or influence others, while position reflected the degree of support or resistance to the GFF-related processes.

Stakeholders were then plotted on a 2x2 grid (high/low power vs. supportive/opposed position), allowing us to visualise who had the most power and interest and the degree to which they were involved in the process (only one document; both documents; or neither).

**Figure S2: 1: Stakeholder Power–Position Grid Analysis for Mozambique GFF policy processes**
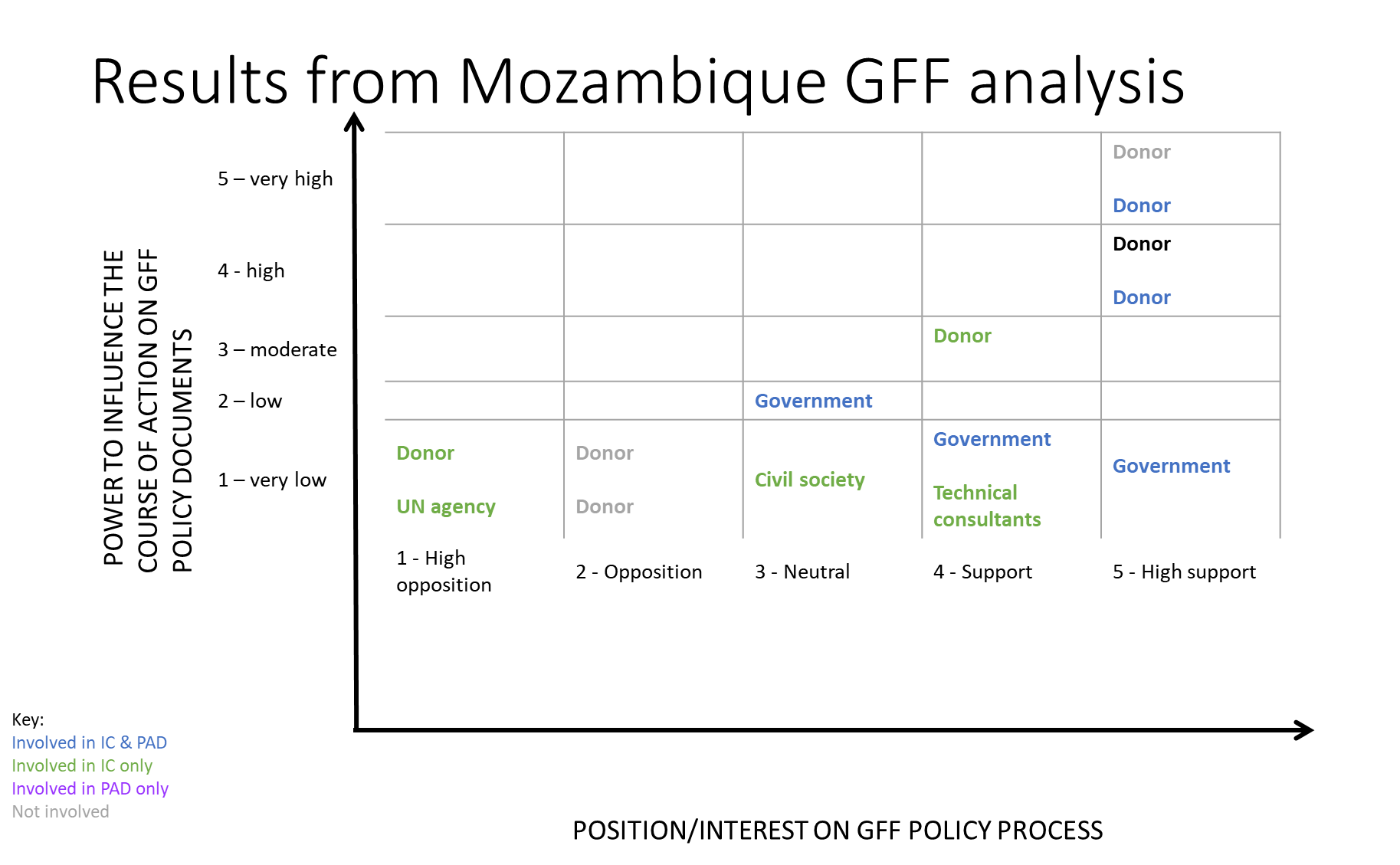

Supplement: Supplementary_files_PRE EXPORT.docx [file ZGHA_A_2518651_SM3356.docx]
